# C3 exoenzyme impairs cell proliferation and apoptosis by altering the activity of transcription factors

**DOI:** 10.1007/s00210-016-1270-2

**Published:** 2016-06-28

**Authors:** Leonie von Elsner, Sandra Hagemann, Ingo Just, Astrid Rohrbeck

**Affiliations:** Institute of Toxicology, Hannover Medical School, Straße 1, D-30625 Hannover, Germany

**Keywords:** C3 exoenzyme, RhoA, Proliferation, Apoptosis, Transcription factors

## Abstract

C3 exoenzyme from *C. botulinum* is an ADP-ribosyltransferase that inactivates selectively RhoA, B, and C by coupling an ADP-ribose moiety. Rho-GTPases are involved in various cellular processes, such as regulation of actin cytoskeleton, cell proliferation, and apoptosis. Previous studies of our group with the murine hippocampal cell line HT22 revealed a C3-mediated inhibition of cell proliferation after 48 h and a prevention of serum-starved cells from apoptosis. For both effects, alterations of various signaling pathways are already known, including also changes on the transcriptional level. Investigations on the transcriptional activity in HT22 cells treated with C3 for 48 h identified five out of 48 transcription factors namely Sp1, ATF2, E2F-1, CBF, and Stat6 with a significantly regulated activity. For validation of identified transcription factors, studies on the protein level of certain target genes were performed. Western blot analyses exhibited an enhanced abundance of Sp1 target genes p21 and COX-2 as well as an increase in phosphorylation of c-Jun. In contrast, the level of p53 and apoptosis-inducing GADD153, a target gene of ATF2, was decreased. Our results reveal that C3 regulates the transcriptional activity of Sp1 and ATF2 resulting downstream in an altered protein abundance of various target genes. As the affected proteins are involved in the regulation of cell proliferation and apoptosis, thus the C3-mediated anti-proliferative and anti-apoptotic effects are consequences of the Rho-dependent alterations of the activity of certain transcriptional factors.

**Electronic supplementary material:**

The online version of this article (doi:10.1007/s00210-016-1270-2) contains supplementary material, which is available to authorized users.

## Introduction

C3 exoenzyme from *Clostridium botulinum* (C3) belongs to the group of eight bacterial ADP-ribosyltransferases including C3lim from *Clostridium limosum*, C3stau from *Staphylococcus aureus*, C3cer from *Bacillus cereus,* and C3larvin from *Paenibacillus larvae* that possess low molecular weight Rho-GTPases as substrates (Aktories and Frevert 1987; Just et al. 1992a; Just et al. 1992b; Wilde et al. 2001; Krska et al. 2015). C3 selectively inactivates the Rho-GTPases RhoA, B, and C by transferring an ADP-ribose moiety from NAD^+^ onto asparagine 41 of Rho (Chardin et al. 1989; Sekine et al. 1989). This resulting loss of functional Rho causes cellular consequences such as disorganization of the actin cytoskeleton, morphological changes, and impaired formation of contractile ring (Wiegers et al. 1991; Kishi et al. 1993). Because of its specificity, C3 is often applied as a selective Rho inhibitor in studying cellular RhoA signaling. Furthermore, the treatment of murine primary hippocampal neurons with C3 reveals an increased axonal growth as well as branching independently of the enzyme activity and an additional dendritotrophic effect of the C3 wild type (Ahnert-Hilger et al. 2004). Moreover, previous studies demonstrated that Rho inactivation by C3 inhibits cell growth in various cell types (Nishiki et al. 1990; Yamamoto et al. 1993; Zuckerbraun et al. 2003; Rohrbeck et al. 2012). RhoA is associated with the regulation of various proteins involved in the control of cell cycle progression like cyclin D1 and p21 (Adnane et al. 1998; Watts et al. 2006). Additionally, RhoA modulates the activity of certain transcription factors known to play a major role in the regulation of cell proliferation. For example, the overexpression of constitutively active RhoAQ63L increases the transcriptional activity of AP-1 and E2F in NIH3T3 cells (Berenjeno et al. 2007). Interestingly, in murine hippocampal HT22 cells, both C3 and enzyme-deficient C3-E174Q mediate inhibition of proliferation that was accompanied by a reduced level of cyclin D1 and increased expression of negative cell cycle regulator RhoB (Du and Prendergast 1999; Rohrbeck et al. 2012).

Besides the inhibition of cell proliferation, previous studies described an influence of C3 on apoptosis in various cell types. Depending on the cell type, C3 is able to trigger apoptosis in EL4 T lymphoma, HUVEC, and hepatic stellate cells (Moorman et al. 1996; Li et al. 2002; Ikeda et al. 2003). Contrary, treatment of astrocytes with C3 after induction of apoptosis with thrombin increases the amount of surviving cells (Donovan et al. 1997). Furthermore, the in vivo application of C3 protects retinal ganglion cells from apoptosis induced either after optic nerve injury or by injection of NMDA (Bertrand et al. 2005; Wang et al. 2014). The injection of C3 on the lesion site decreases the number of apoptotic cells after a spinal cord injury in rodents (Dubreuil et al. 2003). Rohrbeck et al. reported that the prevention of serum-starved and staurosporin-treated HT22 cells from apoptosis is accompanied by the C3-mediated reduction of pro-apoptotic proteins and of the activity of various caspases. Indeed, this anti-apoptotic effect depends on Rho because enzyme-deficient C3-E174Q is without effect (Rohrbeck et al. 2012).

In the present study, we investigated the impact of C3 on the transcriptional level and downstream proteins in HT22 cells. These conditions were selected due to the appearance of C3-mediated inhibition of cell proliferation after 48 h. We demonstrated that C3 Rho-dependently modulated the activity of transcription factors as well as the protein abundance of certain target genes that were associated with the regulation of cell proliferation and apoptosis. Thus, these results strongly indicate that the C3-mediated anti-proliferative and anti-apoptotic effects are mediated by alterations of transcriptional and protein level as a consequence of Rho inactivation by C3.

## Materials and methods

### Cell culture

The murine hippocampal cell line HT22 was cultivated in Dulbecco’s modified essential medium ((Gibco, Life Technologies, Paisley, UK), 10 % fetal bovine serum (PAN Biotech GmbH, Aidenbach, Germany), 1 % penicillin, 1 % streptomycin (PAA Laboratories GmbH, Pasching, Austria), and 1 mM sodium pyruvate (Biochrom AG, Berlin, Germany)) at 37 °C and 5 % CO_2_. When the cells reached confluence, they were passaged.

### Growth kinetics

For growth kinetics experiments, 30,000 cells∙mL^−1^ were seeded onto 3.5-cm plates. After 24 h, the cells were treated with 500 nM C3, C3-E174Q, or 20 nM skepinone-L. Every 48 h the medium was replaced including C3 or C3-E174Q. The determination of cell number was performed as described previously (Rohrbeck et al. 2012).

### qRT-PCR

The isolation of RNA, primer design, and determination of gene expression level of p21 by the use of real-time qRT-PCR measurements were accomplished as described prior (Rohrbeck et al. 2012). The following primer pairs were applied for qRT-PCR: p21/Cdkn1 (NM_007669.4) forward: GTACTTCCTCTGCCCTGCTG; reverse: GGCACTTCAGGGTTTTCTC, B2M (NM_009735.3) forward: ATTCACCCCCACTGAGACTG; reverse: GCTATTTCTTTCTGCGTGCAT. PCR primers were acquired by Eurofins (Ebersberg, Germany).

### Western blot analysis

The cells were seeded onto 3.5-cm plates with a concentration of 150,000 cells∙mL^−1^. The next day, cells were treated with 500 nM C3, C3-E174Q, or indicated concentrations of inhibitors NSC23766 and skepinone-L (Calbiochem, Merck KGaA, Darmstadt, Germany) for various incubation times. After termination of incubation, the cells were washed with ice-cold PBS and frozen at −20 °C. Preparation of cell lysates and Western blot analysis was performed as described previously (Rohrbeck et al. 2012). For the analysis of phosphorylated proteins, 1 mM sodium-ortho-vanadate (Sigma-Aldrich Chemie GmbH, Munich, Germany) was applied in lysis buffer. The following primary antibodies were applied for immunoblotting: α-RhoA, α-p38, α-JNK1, α-p21, and α-GADD153 (Santa Cruz Biotechnology, CA, USA); α-β-Actin (Sigma-Aldrich, St. Louis, MO, USA); and α-pp38 Thr180/182, α-p-c-Jun Ser63, α-COX-2, and α-p53 (Cell Signaling Technology, Beverly, MA, USA). The chemiluminescence reaction was performed by ECL Femto (Pierce, Thermo Fisher Scientific Inc., Rockford, IL, USA), and the signals were detected and analyzed densitometrical by Kodak 1D software (KODAK GmbH, Stuttgart, Germany).

### TF activation profiling plate array

For screening the transcriptional activity of 48 different transcription factors after treatment with C3 for 48 h, the TF Activation Profiling Plate Array I (Signosis Inc., Santa Clara, CA, USA) was performed. HT22 cells were incubated with 500 nM C3 or medium for control conditions. After 48 h, the nuclear extraction (Signosis Inc., Santa Clara, CA, USA) was performed according to manufacturers’ instructions. The protein concentration was determined by Bradford assay, and 5 μg of nuclear extracts per condition were applied in TF Activation Profiling Plate Array I according to manufacturers’ instructions. Both conditions were measured on one 96-well plate containing two sets for each 48 transcription factors. The luminescence was detected at Synergy4 microplate reader (BioTek Instruments Inc., Winooski, VT, USA). For each condition, the relative light units of the transcription factors were normalized to the value of the non-regulated SATB1 as internal control. The relative regulation was calculated by the ratio of C3-treatment in comparison to control condition. Significant regulations were estimated in a twofold increase or decrease of transcriptional activity. Transcription factors whose activity was altered in all three experiments significantly in the same direction were defined as regulated.

### Luciferase reporter experiments

The dual-luciferase reporter experiments were performed with the Cignal Reporter Assay Kit (Qiagen, Hilden, Germany). The reporter system consists of a firefly luciferase reporter under the control of an inducible basal TATA box promotor, with upstream tandem repeat elements (TRE)-sequences for Sp1, and as an internal control, a construct that constitutively expressed *Renilla* luciferase under the control of a CMV immediate early enhancer/promotor in a ratio of 40:1. For detection of background signals, a negative control construct that encodes the firefly luciferase under a non-inducible basal TATA box promotor and a constitutively expressed *Renilla* luciferase (in a ratio of 40:1) were applied. 7500 HT22 cells per well were seeded into 96-well plates. The cells were transfected with 1 μg DNA construct of either transcription factor reporter or negative control by the use of jetPrime Polyplus transfection system (Polyplus transfection S.A., Illkirch, France) according to manufacturers’ instructions. After 4 h, the cells were treated with 500 nM C3, 500 nM C3-E174Q, 20 nM skepinone-L, or 50 μM NSC23766 for 48 h. To attain a stimulation of Sp1 activity, cells were incubated with 100 ng/mL PMA (Sigma-Aldrich Chemie GmbH, Munich, Germany) for 18 h as a positive control. The luciferase activity was determined by Dual-Glo® Luciferase assay system (Promega Corporation, Madison, WI, USA) on Synergy4 microplate reader (BioTek Instruments Inc., Winooski, VT, USA). Data were processed by normalizing the relative light units of firefly to *Renilla* luciferase, subtracting background signals and calculating the relative regulation of transcriptional activity. To determinate the effectivity of transfection, cells were transfected with a positive control reporter containing a construct that encodes GFP. The cells were visualized by light and fluorescence microscopy (Zeiss Axiovert 200 M; Carl Zeiss GmbH, Göttingen, Germany).

### Expression and purification of recombinant C3 proteins

C3 wild type and C3-E174Q were expressed as recombinant fusion proteins with a glutathione S-transferase (GST)-tag into plasmid pGEX-2T (gene of *C. botulinum* C3, accession no. X59039) that was transferred into *E. coli* TG1. The purification of recombinant protein was performed as described previously (Rohrbeck et al. 2012).

### Reproducibility of the experiments and statistics

All experiments were performed independently at least three times. The figures display results from representative experiments. For graphical and statistical analysis, Microsoft® Excel 2010 version 14.0 (Microsoft Corporation, Redmond, USA) was applied. The values (*n* ≥ 3) are means ± SEM. The statistical significance of differences between treated compared to untreated cells were calculated by the use of a two-sided unpaired Student’s *t* test (* = *p* ≤ 0.05, ** = *p* ≤ 0.01, and *** = *p* ≤ 0.001). The statistical differences between treated compared to untreated cells in qRT-PCR experiments were calculated by the use of a one-sided unpaired Student’s *t* test (* = *p* ≤ 0.05).

## Results

### C3 altered the transcriptional activity of ATF2 and Sp1

**Fig. 1:**
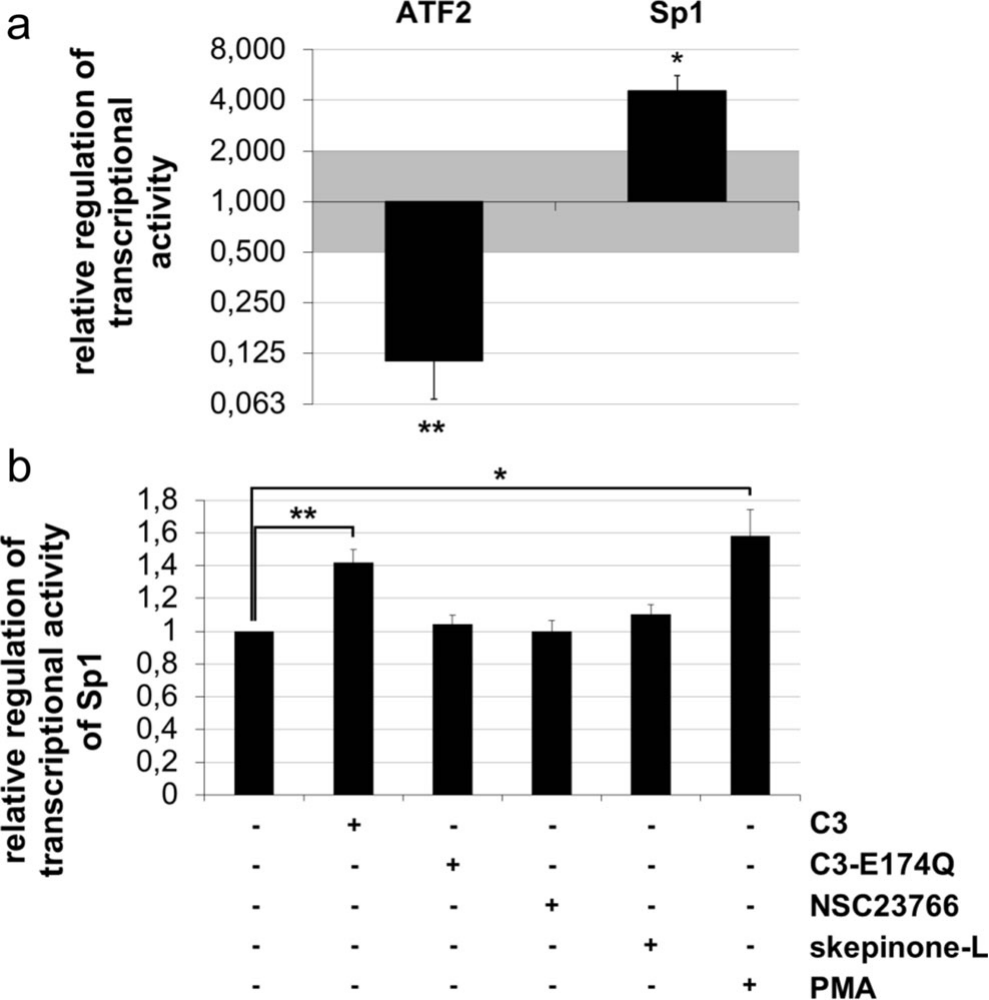
C3-mediated modifications of the activity of various transcription factors. **a** The nuclear extracts of HT22 cells incubated with 500 nM C3 or control medium for 48 h were prepared and applied in TF Activation Profiling Plate Array I. For data analysis, the relative light units of the transcription factors were normalized to the value of the non-regulated transcription factor SATB1 as internal control and the relative regulation of transcriptional activity were determined by comparison of C3-treated to untreated cells. A significant change in transcriptional activity was assumed by twofold upregulation or downregulation. Exemplarily, the relative regulation of ATF2 and Sp1 was depicted with the mean values ± SEM of three independent experiments (*n* = 3). **b** HT22 cells were transfected with luciferase reporter constructs and treated with 500 nM C3, 500 nM C3-E174Q, 50 μM NSC23766, or 20 nM skepinone-L for 48 h. As a positive control, the cells were incubated with 100 ng/mL PMA for 18 h. The relative regulation of transcriptional activity of Sp1 was determined by the ratio of treated to untreated cells. Mean values ± SEM are illustrated of at least three experiments

To get an overview of those transcription factors influenced by C3, the TF Activation Profiling Plate Array I was carried out for simultaneously analyzing 48 different transcription factors. Data analysis of this array exhibited five transcription factors, namely Sp1, ATF2, CBF, E2F-1, and Stat6, that were significantly regulated in all three independent experiments as shown in Table S1. For this study, we focused on the influence of C3 on Sp1 and ATF2, as examples for an upregulation and downregulation, respectively, of transcription factors. Both transcription factors are essentially involved in the regulation of numerous cellular processes, such as cell proliferation and apoptosis (Walton et al. 1998; Deniaud et al. 2009). As summarized in Fig. 1a, the transcriptional activity of ATF2 was distinctly reduced, while the activity of Sp1 increased after incubation with C3 for 48 h. To verify exemplarily, the results of the upregulated transcription factor Sp1, a luciferase reporter assay, was applied. The enhanced transcriptional activity of Sp1 after treatment with C3 for 48 h was confirmed by the luciferase assay, whereas the enzyme-deficient C3-E174Q did not influence the activity of Sp1 (Fig. 1b). To get an idea of further participating signaling pathways, the Rac inhibitor NSC23766 was applied. NSC23766 is known to selectively inhibit Rac1 by impairing the activation of the Rac-specific GEFs Tiam1 and Trio (Gao et al. 2004). Notably, no effect was detectable after the incubation of HT22 cells with the Rac inhibitor under the chosen conditions. Additionally, experiments with the p38 inhibitor skepinone-L were performed. Skepinone-L is the first highly selective ATP-competitive p38 inhibitor that was identified in 2011 by Koeberle et al. (Koeberle et al. 2012a; Koeberle et al. 2012b). Interestingly, skepinone-L only enhanced marginally the transcriptional activity of Sp1 (Fig. 1b) in the luciferase assay. As positive control cells were incubated with 100 ng/mL PMA for 18 h that increased the Sp1 activity by 1.6-fold. For the determination of transfection effectivity, cells were transfected with a supplied positive control reporter that contains an additional construct encoding for GFP (Fig. S1). A convincing effectivity was detected by fluorescence microscopy of GFP-transfected cells. These consistent results of both assays revealed a C3-mediated alteration of the transcriptional activity of Sp1.

For validation of the identified transcription factors, the downstream target genes of Sp1 and ATF2 were analyzed. Western blot analyses of the three different Sp1 target genes p21, c-Jun, and cyclooxygenase (COX)-2 harboring at least one Sp1 binding site in their gene promotor were performed (Rozek and Pfeifer 1993; Appleby et al. 1994; Datto et al. 1995; Rozek and Pfeifer 1995; Biggs et al. 1996; Xu et al. 2000). In case of c-Jun, we focused on the activation in terms of a phosphorylation of c-Jun. The enzyme-deficient C3-E174Q was carried along as a negative control, as it did not provoke any alterations in transcriptional activity of Sp1. Additionally, the effects of Rac and p38 inhibitors on the target proteins were determined.

### C3-induced increase of p21 and anti-proliferative effect

**Fig. 2:**
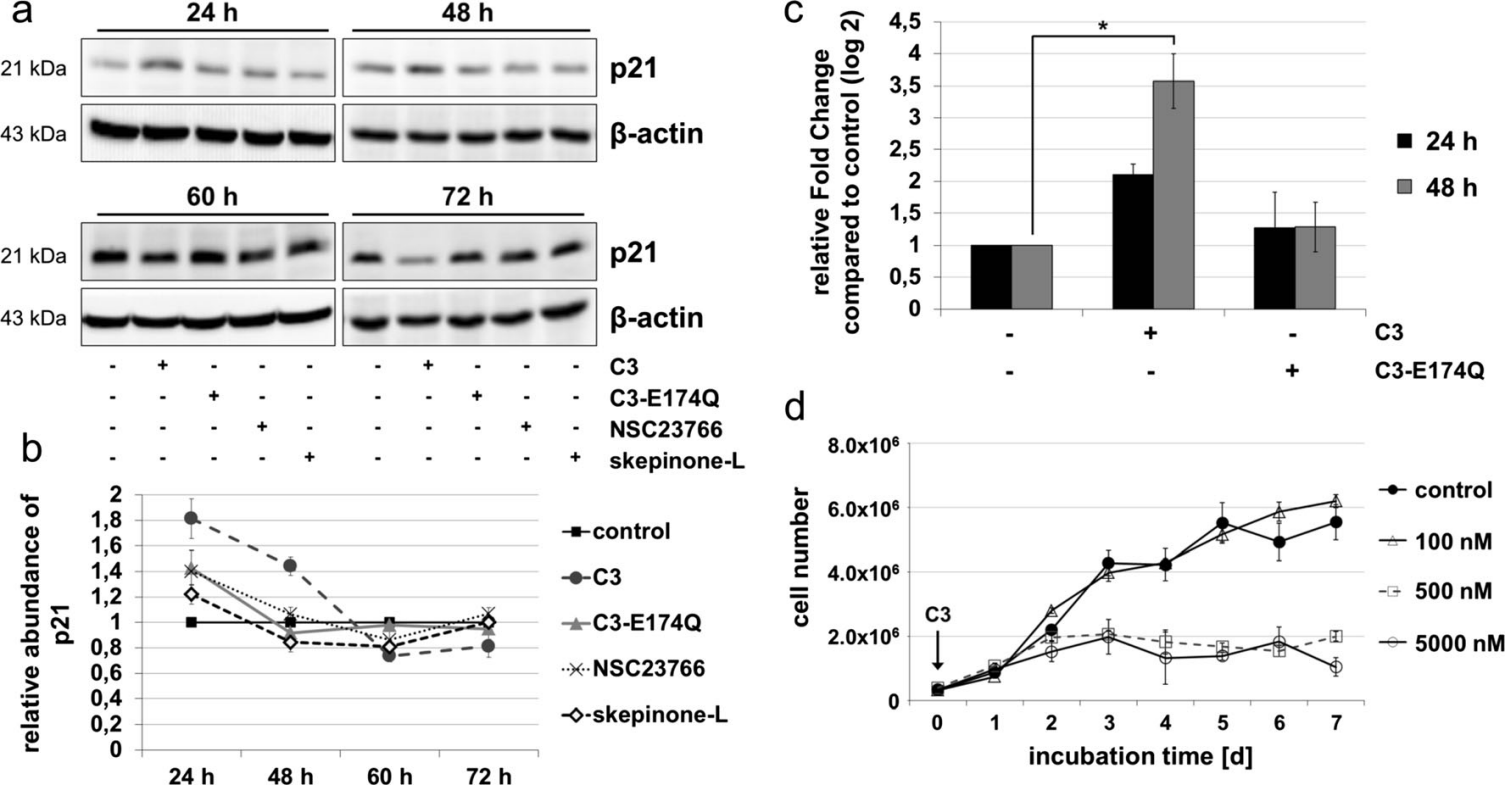
Influence of C3 on p21 and C3-mediated effects on cell proliferation. **a** HT22 cells were incubated with 500 nM C3, 500 nM C3-E174Q, 50 μM NSC23766, or 20 nM skepinone-L for indicated time points. Cells were lysed and applied to Western blot analysis for p21 and β-actin. Western blots from representing experiments are shown. **b** Relative abundance of p21 (mean values ± SEM) were calculated by normalizing the signal intensity of p21 to the corresponding intensity of β-actin and comparing treated to untreated cells of three independent experiments (*n* = 3). **c** HT22 cells were treated with 500 nM C3 and C3-E174Q for 24 and 48 h. The isolated RNA was applied in quantitative RT-PCR to determine the gene expression of p21. The ΔCt value of p21 was normalized to ΔCt of house-keeping gene β2-microglobulin. Results represent mean values ± SEM of three independent experiments (*n* = 3). **d** To investigate the concentration dependence of C3-mediated inhibition of cell proliferation, growth kinetic experiments were performed. HT22 cells were treated with 100, 500, and 5000 nM C3 by replacing the medium including C3 every 48 h. At indicated time points, the cell number was determined by trypan blue counting assay in triplicate. Growth curves illustrate mean values ± SEM

C3 increased the protein abundance of p21 significantly after 24 h. After 48 h, the abundance of p21 was still enhanced compared to the control. From 60 h on C3, the level of p21 is reduced by approximately 25 % (Fig. 2a, b). RT-PCR data shown in Fig. 2c confirmed that C3 also raised gene expression of p21 by twofold after 24 h and by 3.5-fold after 48 h. C3-E174Q raised the protein abundance of p21 after 24 h but did not provoke any detectable effect neither on protein nor on mRNA level at the other incubation times. The p38 inhibitor skepinone-L decreased p21 by about 15–20 % after 48 and 60 h.

As p21 is a major regulator of cell cycle progression; the effect of C3 on cell proliferation was examined by growth kinetic experiments in a concentration-dependent manner (Fig. 2d). Incubation with 100 nM of C3 did not impair the proliferation of HT22 cells. A fivefold raise of the concentration of C3 caused an inhibition of cell growth from the second day of treatment. Interestingly, a tenfold increase in concentration to about 5 μM of C3 did not further enhance the observed proliferation inhibition shown in Fig. 2d nor resulted in any signs of cellular toxicity. The temporal delay of 48 h of the C3-mediated inhibition of cell proliferation was independently of the cellular growth phase and only correlated with the incubation time with C3 (Fig. S2). In contrast, the enzyme-deficient C3-E174Q induced a Rho-independent, medium inhibition of cell proliferation in HT22 cells (Rohrbeck et al. 2012). The p38 inhibitor skepinone-L inhibited the proliferation of HT22 cells moderately starting from the second day of incubation (Fig. S3). A combined incubation of C3 and skepinone-L provoked a minimum increased anti-proliferative effect compared to the single C3 treatment confirming the involvement of p38 in the C3-mediated anti-proliferative effect.

### C3 induced increase in phosphorylation of c-Jun and reduced the level of p53

**Fig. 3:**
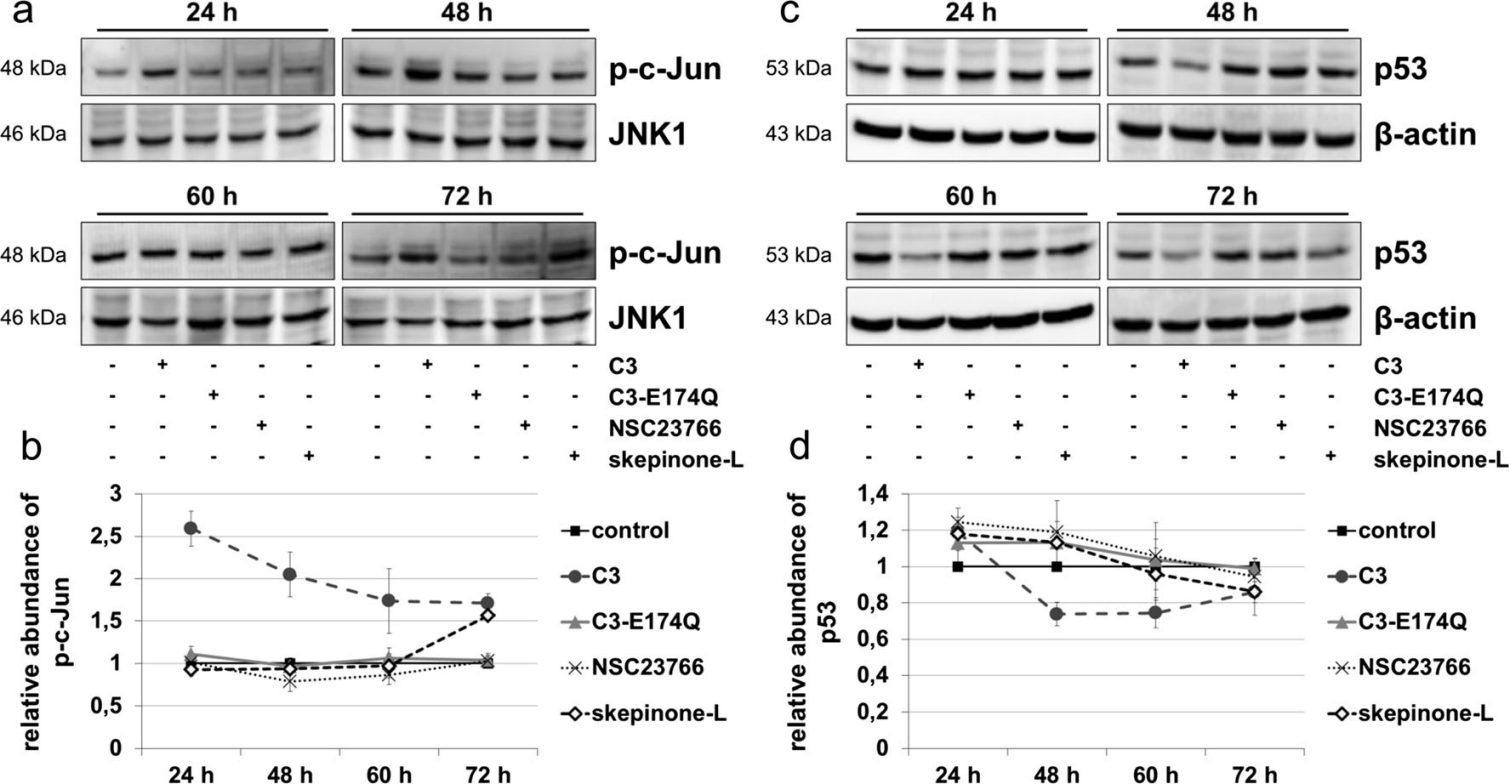
Influence of C3 on the phosphorylation of c-Jun and the abundance of p53. **a** HT22 cells were treated with 500 nM C3, 500 nM C3-E174Q, 50 μM NSC23766, or 20 nM skepinone-L for indicated time points, lysed, and applied to Western blot analysis for phospho-c-Jun (p-c-Jun) and c-Jun N-terminal kinase 1 (JNK1). **b** Densitometric quantifications were performed by adjusting the signal intensity of p-c-Jun to the corresponding intensity of JNK1. The same experimental procedure of (**a**) and (**b**) was applied for Western blot analysis (**c**) and densitometric quantification (**d**) of p53 and β-actin. Representing Western blot analyses are illustrated. Results represent mean values ± SEM of independent experiments of p-c-Jun (*n* = 3) and p53 (*n* = 3)

Western blot analysis of phospho-c-Jun was applied to study the impact of C3 on the activation of c-Jun. The abundance of phospho-c-Jun was increased significantly by approximately 2.5-fold after treatment with C3 for 24 and decreased to 1.7-fold over time (Fig. 3a, b). The enzyme-deficient C3-mutant did not exhibit any effect on the phosphorylation of c-Jun, whereas skepinone-L enhanced significantly the level of phospho-c-Jun after 72 h. To determine the functionality of activated c-Jun, the cell cycle regulator p53, a downstream target gene of c-Jun, was analyzed. c-Jun is involved in the regulation of various cellular processes, including the regulation of p53 via an AP-1-like site, namely PF-1 site, in the p53 promotor (Ginsberg et al. 1990; Schreiber et al. 1999). After 24 h, the abundance of p53 was raised insignificantly by C3, C3-E174Q, and both inhibitors. C3 reduced the level of p53 by 25 % from 48 h on and maintained a reduction of 15 % up to 72 h (Fig. 3c, d). C3-E174Q slightly increased the abundance of p53 until 48 h, whereas skepinone-L first marginally increased the level of p53 after 48 h and then reduced the abundance comparably to C3 after 72 h.

### C3 modulated the level of COX-2 biphasically

**Fig. 4:**
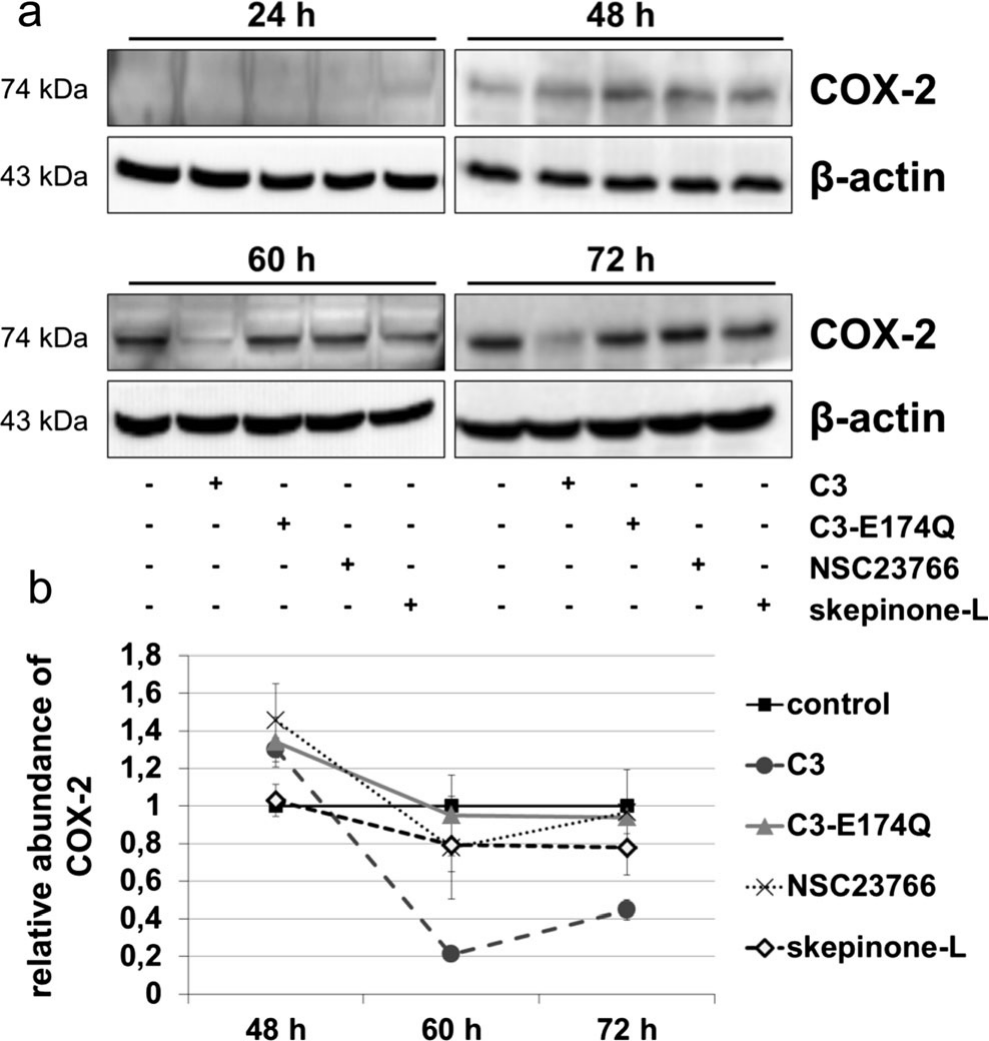
C3-induced effects on the abundance of COX-2. **a** Cells were incubated with 500 nM C3, 500 nM C3-E174Q, 50 μM NSC23766, or 20 nM skepinone-L for indicated time points. Cell lysates were submitted to Western blot analysis for COX-2 and β-actin. Representing blots are shown. **b** Data analysis was determined by normalization of the signal intensity of COX-2 to the corresponding intensity of β-actin. Mean values ± SEM are illustrated of four independent experiments (*n* = 3)

The level of COX-2 was elevated up to 30 % in cells treated with C3 and C3-E174Q and by 40 % after treatment with NSC23766 for 48 h (Fig. 4a, b).

Surprisingly, C3 reduced distinctly and significantly the abundance of COX-2 by more than 60 % after 60 and 72 h. Incubation with skepinone-L from 60 h on lowered slightly the COX-2 level by 20 %, but no effect was detectable after treatment with C3-E174Q for 60 and 72 h. The decrease in COX-2 starting from 60 h revealed a biphasic modulation mediated by C3. Due to the weak signal intensity at Western blot analysis, the quantification of COX-2 after 24 h was not reliable.

Taken collectively, the increased protein abundances of p21, phospho-c-Jun, and COX-2 after treatment with C3 for 48 h indicated an enhanced activity of Sp1.

### C3-induced alterations in p38 activity and reduction of GADD153

**Fig. 5:**
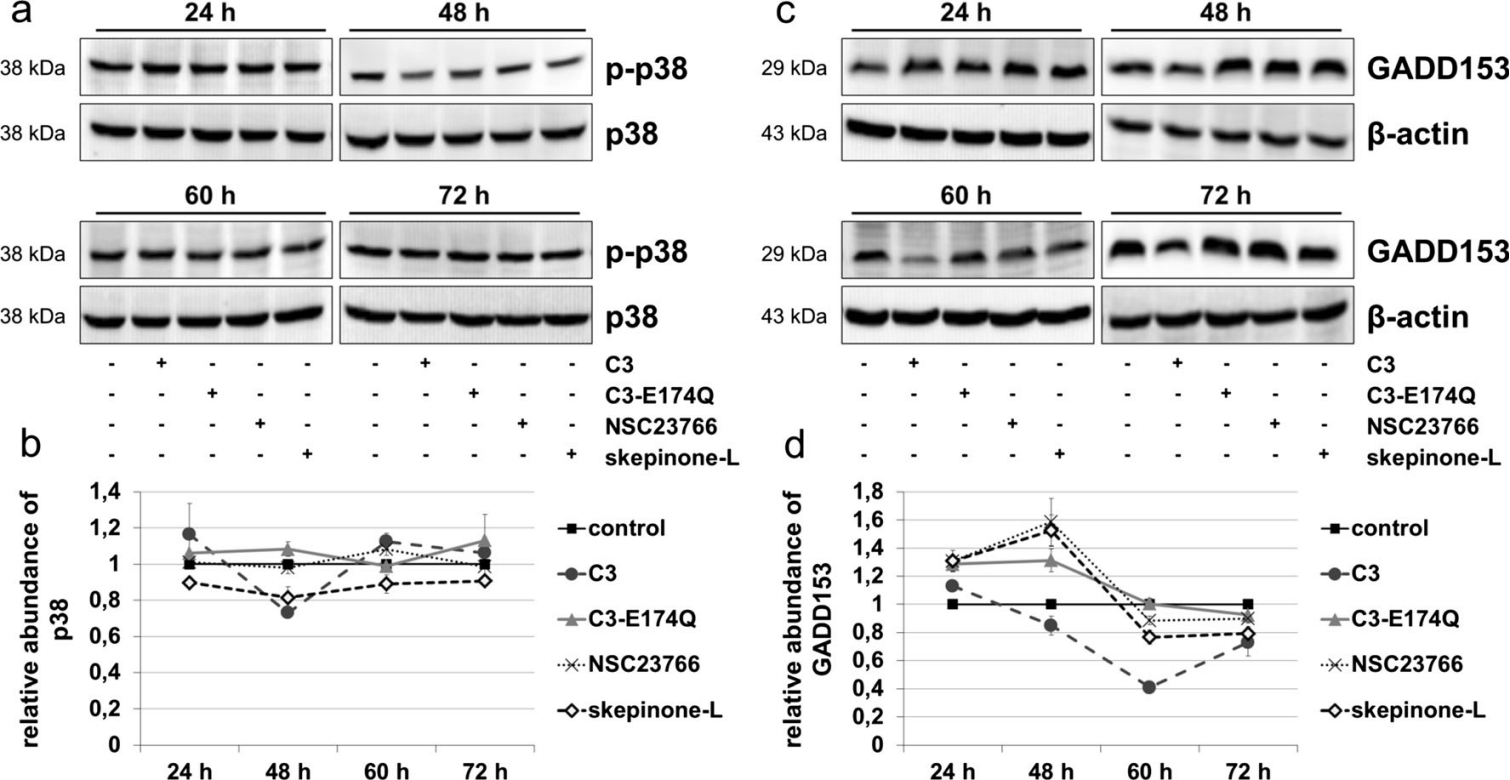
C3-mediated changes of phosphorylation status of p38 and reduction of GADD153. **a** HT22 cells were treated with 500 nM C3, 500 nM C3-E174Q, 50 μM NSC23766, or 20 nM skepinone-L for indicated time points. Cells were lysed and applied to Western blot analysis for phospho-p38 and total p38. **b** For densitometric analysis, the signal intensity of phosphorylated p38 was adjusted to the corresponding intensity of p38. The same experimental procedure of (**a**) and (**b**) was applied for Western Blot analysis (**c**) and densitometric quantification (**d**) of GADD153 and β-actin. Representing Western blot analyses are shown. Results depict mean values ± SEM of independent experiments of p38 (*n* = 3) and GADD153 (*n* = 3)

With regard to the C3-mediated anti-apoptotic effect, the signaling cascade of ATF2, which is associated with apoptosis induction, was analyzed (Walton et al. 1998). The activity of ATF2 is regulated via phosphorylation by various kinases including p38 MAP kinase (Raingeaud et al. 1996). Therefore, the influence of C3 on the phosphorylation of p38 was studied by Western blot analysis (Fig. 5a, b). The level of phospho-p38 was significantly decreased by more than 25 % after incubation with C3 for 48 h but was not significantly altered in C3-treated cells after 60 and 72 h. In contrast, C3-E174Q did not affect phospho-p38 until 60 h but led to a marginal increase of phospho-p38 after 72 h. Skepinone-L reduced continuously the abundance of phospho-p38 from 24 h on. The Growth Arrest and DNA Damage-inducible protein 153 (GADD153) is involved in the induction of apoptosis and is regulated by ATF2 via the p38 MAP kinase pathway (Bruhat et al. 2000; Maytin et al. 2001; Oh-Hashi et al. 2001; van der Sanden et al. 2004). Western blot analysis exhibited a reduced abundance of GADD153 after treatment with C3 starting from 48 h (Fig. 5c, d). The level of GADD153 was raised by 30–50 % in cells treated with C3-E174Q, NSC23766, and skepinone-L for 24 and 48 h. However, furthermore, we only focused on the inhibitory effects of skepinone-L in detail due to the fact that the p38 signaling pathway was more promising as described in the literature to be involved in the regulation of ATF2 (Raingeaud et al. 1996). After treatment with skepinone-L for 60 and 72 h, the abundance of GADD153 was decreased significantly by 20 %, whereas C3-E174Q provoked only minimum effects at this time. These results indicated a C3-mediated effect on p38 MAPK signaling that might lead downstream via ATF2 to a decreased abundance of GADD153.

## Discussion

**Fig. 6:**
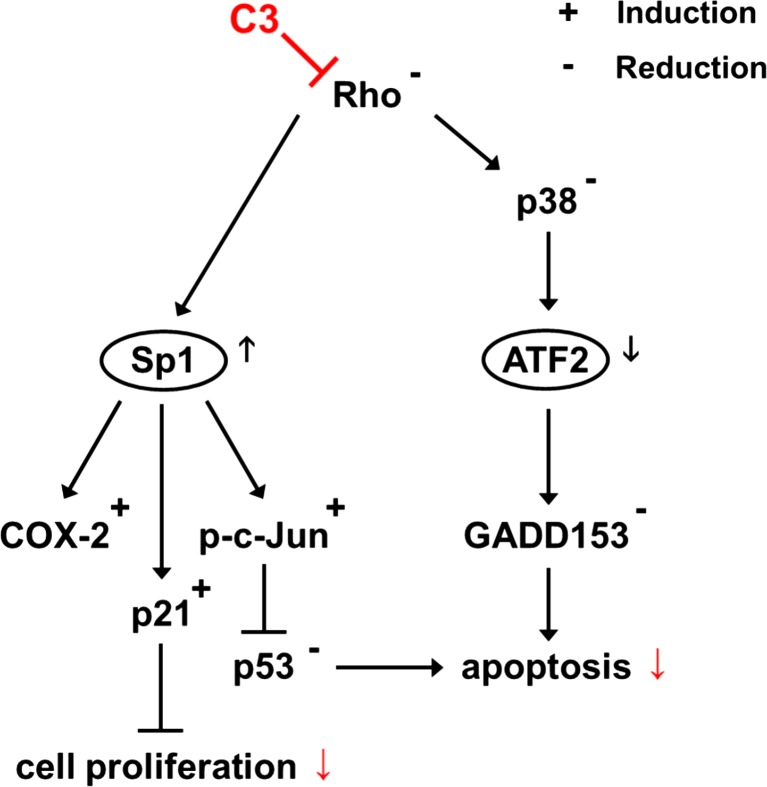
Proposed signaling pathways involved in C3-mediated anti-proliferative and anti-apoptotic effects. As a consequence of Rho inactivation by C3, the transcriptional activities of Sp1 and ATF2 are modulated resulting downstream in altered abundances and activities of the target genes involved in the regulation of proliferation and apoptosis. The transcription factor Sp1 regulates the abundance of p21, COX-2, and p-c-Jun. In turn, c-Jun is a regulator of the level of p53, whereas p21 is an inhibitor of cell cycle progression. RhoA is able to regulate the activity of MAPK p38 via downstream signaling cascades. However, p38 is an activator of ATF2 that in turn regulates the abundance of GADD153. Both p53 and GADD153 are inducers of apoptosis

Because of its selective inactivation of Rho-GTPases, C3 is a biological tool to study the involvement of RhoA, B, and C in cellular processes. Previous studies have already revealed that the C3-mediated ADP-ribosylation of RhoA results in an inactivation and degradation of RhoA in various cell types (Rohrbeck et al. 2012; Rohrbeck et al. 2015a, b). In HT22 cells treated with C3 for 48 and 72 h, a large percentage of the cellular RhoA is degraded as detected by a distinct reduction of RhoA in Western blot analysis (Rohrbeck et al. 2012; Rohrbeck et al. 2015b). But until now, only less is known about the impact of C3 on Rho downstream pathways effecting cell proliferation and apoptosis. In the present study, we identified five transcription factors that were regulated in hippocampal HT22 cells in response to C3. With regard to the described C3-mediated anti-proliferative and anti-apoptotic effects, it is not surprising that the identified transcription factors Sp1, ATF2, and E2F are major regulators of cell proliferation and apoptosis (Mudryj et al. 1990; Shirodkar et al. 1992; Walton et al. 1998; Deniaud et al. 2009). Additionally, also Stat6 and CCAAT/enhancer binding protein zeta (CBF) are involved in the regulation of cell cycle-dependent genes (Milarski and Morimoto 1986; Lum et al. 1990; Kaplan et al. 1998). However, RhoA is involved in the regulation of various transcription factors like AP-1 and E2F (Rivard et al. 1999; Berenjeno et al. 2007). Consistent with our results, overexpression of constitutively active RhoAQ63L increases the transcriptional activity of E2F, whereas C3 inhibits serum-stimulated E2F activity (Rivard et al. 1999; Berenjeno et al. 2007). Constitutively active RhoAQ63L also triggers AP-1 activity (Berenjeno et al. 2007). In contrast, C3 does not impair AP-1 activity in cardiac muscle cells after stimulation of AP-1 promotor activity with phenylephrine (Thorburn et al. 1997). These results are in agreement with our data that AP-1 is not regulated by C3. The present study focused on C3-induced alterations of the activity of Sp1 and ATF2, since E2F, CBF, and Stat6 are subjects of separate studies because of the broad spectrum of regulated downstream pathways. The missing effect of C3-E174Q on Sp1 activity strongly indicates that C3 modulates the activity of Sp1 via Rho and related downstream cascades like MAP kinase signaling (Fig. 6). In this context, the impact of p38 can be neglected because the reduction of p38 activity by Rho inhibitor C3 and the p38 inhibitor skepinone-L did not affect Sp1 activity. To further verify the identified transcription factors and characterize the extent of C3-mediated effects on protein level, the protein abundances of certain target genes of Sp1 were examined. Especially Sp1 is known for transcriptional regulation of proteins involved in cell cycle control such as c-Jun, p21, and various cyclins (Harper et al. 1993; Wisdom et al. 1999; Deniaud et al. 2009). Previous studies reported that the C3-mediated anti-proliferative effect after 48 h is featured by the inactivation and degradation of ADP-ribosylated RhoA, an enhanced expression of RhoB and a decrease in cyclin D1 (Rohrbeck et al. 2012). Now, in this study, we determined an enhanced activity of Sp1 accompanied by an altered protein abundance of its downstream target genes p21, phospho-c-Jun, and COX-2 at this time. In agreement with previous studies, C3 increased the level of p21 on RNA and protein level after 24 and 48 h. After treatment of smooth muscle cells with C3 for 24 h, the protein level of p21 is raised and the activity of p21 promotor is enhanced by C3 in various cell lines (Adnane et al. 1998; Zuckerbraun et al. 2003). Besides p21, c-Jun is a major player of regulation of cell proliferation that was phosphorylated distinctly and continuously after treatment with C3 starting from 24 h in this current work. A functional connection between overexpression of Sp1 and an increased level of c-Jun was first described in murine IL-3-dependent Baf-3 cells. These cells overexpresses Sp1 after induction with doxycycline exhibiting a rise in c-Jun expression detected by microarray analysis and RT-PCR (Deniaud et al. 2009). In accordance with our results, Alberts & Treisman described an increased phosphorylation of c-Jun after transfection of NIH 3T3 cells with C3 (Alberts and Treisman 1998). To determine the functionality of this activation of c-Jun, the abundance of p53 was examined. The observed decreased abundance of p53 after incubation with C3 for 48 until 72 h is consistent with previous data of serum-starved HT22 cells that showed a reduced level of p53 under similar conditions (Rohrbeck et al. 2012). Ginsberg et al. first described a binding of c-Jun to a PF1-site in the p53 promotor causing an uncommon repression of gene expression (Ginsberg et al. 1990). In accordance with our results, Schreiber et al. reported that the level of p53 is increased in c-Jun-deficient 3T3-fibroblasts in comparison to wild-type cells. Moreover, stable overexpression of c-Jun in those cells reduces the p53 expression. Interestingly, concurrently, the abundance of p21 is increased in the c-Jun-deficient cells and is reduced after overexpression of c-Jun (Schreiber et al. 1999). In contrast to that, Kardassis et al. demonstrated that a simultaneous overexpression of c-Jun and Sp1 transactivates the p21 promotor in *Drosophila* Schneider’s SL2 and HepG2 cells (Kardassis et al. 1999). In this context, it is possible that the simultaneous overexpression of c-Jun and Sp1 exceeded the c-Jun-mediated repression of p21. Taken together, we identified a C3-mediated enhanced Sp1 activity resulting in an increased level of p21 and phosphorylated c-Jun that in turn reduced the level of p53. This is further supported by the missing effects of enzyme-deficient C3-E174Q and the insignificant impact of the Rac and p38 inhibitors on Sp1 and the studied target proteins. Thus, C3 regulated specifically the Sp1 activity via Rho inactivation, whereas the influence of Rac and p38 is negligible. Certainly, the moderate effect of skepinone-L on cell proliferation, the slight enhancement of C3-mediated anti-proliferative effect in combination with C3 in growth kinetic experiments, and the modified levels of phospho-c-Jun after 72 h indicated an influence of p38 on cell proliferation. Due to the missing alterations of Sp1 and target proteins after treatment with skepinone-L until 60 h, this effect plays indeed a minor role in the proposed Sp1 signaling. However, also C3-E174Q exhibits a moderate anti-proliferative effect in HT22 cells indicating an additional Rho-independent impact on cell proliferation (Rohrbeck et al. 2012). Overall, this observed combination of altered signaling pathways is able to cause an inhibition of cell proliferation, mainly based on an increased activity of Sp1 and enhanced abundance of p21 that are highly associated with cell cycle arrest (Harper et al. 1993; Deniaud et al. 2009). The impact of c-Jun on cell proliferation is cell type-dependent, because the growth of c-Jun-deficient embryonic stem cells is not influenced, but c-Jun-deficient fibroblasts are arrested in the G_1_ phase of the cell cycle (Hilberg and Wagner 1992; Wisdom et al. 1999). Our results demonstrated that the interactions of altered levels of Sp1, p21, and phospho-c-Jun play a crucial role in C3-mediated inhibition of cell proliferation. The influence of the other identified transcription factors such as E2F, another major regulator of cell proliferation, is still unclear. Further studies are ongoing to clarify the role.

In a rat spinal cord injury model, the expression of Sp1 target gene COX-2, a marker protein of inflammation, is increased up to 48 h (Appleby et al. 1994; Resnick et al. 1998; Xu et al. 2000). Indeed, the expression of COX-2 is not only induced by inflammation and after injuries but COX-2 is also constitutively expressed on a basal level in neuronal cells of the spinal cord and certain areas of the brain (Yamagata et al. 1993; Resnick et al. 1998). Nevertheless, the enhanced expression after 48 h was mediated enzyme-independently, because both C3 and C3-E174Q induced this effect. The C3-mediated decreased COX-2 abundance after 60 and 72 h is in agreement with previous studies demonstrating that the induction of COX-2 promotor by a constitutively active Gα13-subunit of heteromeric G proteins is blocked in NIH 3T3 cells after transfection with a C3 expression vector for 72 h (Slice et al. 1999). Due to the fact that also the p38 inhibitor skepinone-L slightly reduced COX-2 by 20 %, after 72 h, the p38 signaling seems to play a minor but Sp1-independent role in the regulation of COX-2.

Besides the inhibition of cell proliferation, C3 prevents serum-starved HT22 cells from apoptosis by downregulation of the pro-apoptotic proteins Bax, Bid, p53, and certain caspases at an mRNA and protein level. Moreover, the enzyme activity of caspase-3 and caspase-7 is reduced by C3 treatment for 48 h (Rohrbeck et al. 2012). Among the identified transcriptional factors, especially ATF2 is involved in the transcription of apoptosis-inducing proteins like GADD153, whose increased expression is strongly associated with induction of apoptosis in various cell types (Walton et al. 1998; Bruhat et al. 2000; Maytin et al. 2001; Oh-Hashi et al. 2001; van der Sanden et al. 2004). However, the C3-mediated protection from apoptosis in HT22 cells is in agreement with our findings of a reduced GADD153 abundance. The activation of ATF2 is mediated via phosphorylation by certain kinases such as p38 and JNK (Gupta et al. 1995; Raingeaud et al. 1996). The observed decreased level of phosphorylated p38 supports a connection between ATF2 and p38. The findings are endorsed by a study of Pausawasdi et al. identifying a C3-induced decrease in carbachol-stimulated p38 activity (Pausawasdi et al. 2000). These results imply that the reduced level of phosphorylated p38 may lead downstream to a decreased activity of ATF2. The proposed correlation between p38, ATF2, and GADD153 is further strengthened by the C3-like effects of the p38 inhibitor reducing moderately the level of GADD153 by 20 % after incubation with skepinone-L for 60 and 72 h. In agreement with these findings, prior studies reported that GADD153 transcription is highly associated with p38 in the context of apoptosis induction in various cell types (Oh-Hashi et al. 2001, Wang and Ron 1996). Moreover, GADD153 can also be activated directly by p38 via phosphorylation at serine 78 and 81 (Maytin et al. 2001). Additionally, the missing inhibiting effects of C3-E174Q on phospho-p38 and GADD153 strongly indicate a Rho-dependent reduction of ATF2 activity as a result of the decreased activity of p38 downstream inhibiting the GADD153 abundance (Fig. 6). With regard to the impact of p53 and c-Jun on the C3-mediated anti-apoptotic effect, a prior study in primary hepatocytes demonstrated that c-Jun not only represses the p53 expression via the PF-1 site but also antagonizes p53 activity after apoptosis induction by TNFα (Ginsberg et al. 1990; Schreiber et al. 1999; Eferl et al. 2003). Accordingly, as already mentioned for the C3-induced growth inhibition, also the C3-mediated prevention of apoptosis represents a consequence of the several alterations on the transcriptional and downstream protein level interfering to the anti-apoptotic impact.

In conclusion, we demonstrated that C3-mediated inactivation of Rho-GTPases also influenced transcriptional regulation involved in distinct cellular functions in addition to reorganization of the actin cytoskeleton. We identified a Rho-dependent effect of C3 on transcription factors such as Sp1 and ATF2 and their downstream target genes that were strongly involved in cell proliferation and apoptosis. Thus, these alterations in cell signaling after 48 h result in the C3-mediated anti-proliferative and anti-apoptotic effects.

## Electronic supplementary material

ESM 1 (PDF 541 kb)

## Abbreviations

C3: C3 exoenzyme from *Clostridium botulinum*
C3-E174Q: Enzyme-deficient C3-mutant
PMA: Phorbol-12-myristate-13-acetate
Sp1: Specificity protein 1
ATF2: Activating transcription factor 2
E2F-1: E2F transcription factor 1
CBF: CCAAT/enhancer binding protein (C/EBP), zeta
Stat6: Signal transducer and activator of transcription 6
COX-2: Cyclooxygenase 2
GADD153: Growth Arrest and DNA Damage-inducible protein 153
