# Supplementary material for: C3 exoenzyme impairs cell proliferation and apoptosis by altering the activity of transcription factors
Source: Naunyn Schmiedebergs Arch Pharmacol. 2016 Jun 28;389:1021–31. doi: 10.1007/s00210-016-1270-2 (PMC4977334; doi:10.1007/s00210-016-1270-2)
Supplement: Supplementary file 1 — (PDF 541 kb) [file 210_2016_1270_MOESM1_ESM.pdf]

Naunyn-Schmiedeberg's Archives of Pharmacology
C3 exoenzyme impairs cell proliferation and apoptosis by altering the activity of transcription factors
L. von Elsner, S. Hagemann, I. Just and A. Rohrbeck
Institute of Toxicology, Hannover Medical School, Hannover, Germany
email: vonelsner.leonie@mh-hannover.de

Supplementary Material

Table S1 Identified regulated and non-regulated transcription factors in TF Activation Profiling Plate Array I. The table summarized the results of three independent TF Activation Profiling Plate Arrays. For data analysis the relative light units of the transcription factors were normalized to the value of the non-regulated transcription factor SATB1 as internal control and the relative regulation of transcriptional activity were determined by comparison of C3-treated to untreated cells. A significant change in transcriptional activity was assumed by 2-fold up- or down-regulation. Transcription factors in bold were significantly regulated by C3. Statistical differences between C3-treated compared to untreated cells were determined by use of a two-sided unpaired Student‘s t test (* p ≤ 0.05; ** p ≤ 0.01; *** p ≤ 0.001)

Transcription factor	Gene description	Relative regulation of 3 independent experiments			Mean value	Signi-ficance
AP1	Activator protein 1 (JUN/FOS)	0,4467	0,0014	1,7659		
AP2	Activator protein 2	0,0609	0,0164	3,9586		
AR	Androgen receptor	0,0640	39,1750	0,3754		
**ATF2**	**activating transcription factor 2**	**0,0024**	**0,1567**	**0,1765**	**0,1119**	**
Brn-3	POU domain, class 4, transcription factor 1	0,8053	0,1134	1,6485		
C/EBP	CCAAT/enhancer binding protein (C/EBP), alpha	0,8478	0,0592	3,0478		
CAR	nuclear receptor subfamily 1, group I, member 3	0,5326	0,0085	3,3262		
**CBF**	**CCAAT/enhancer binding protein (C/EBP), zeta**	**0,0440**	**0,1238**	**0,3173**	**0,1617**	**
CDP	cut-like homeobox 1; CCAAT displacement protein	0,4621	0,1560	1,9607		
CREB	cAMP responsive element binding protein 1	0,2838	0,0613	0,5839		
**E2F-1**	**E2F transcription factor 1**	**0,0873**	**0,1450**	**0,1620**	**0,1314**	***
EGR	Early growth response	0,0550	0,0282	1,5332		
ER	Estrogen receptor	5,7465	0,3233	3,0870		
Ets	v-ets erythroblastosis virus E26 oncogene homolog 1	0,0606	0,1278	1,4584		
FAST-	Forkhead box H1	1,1037	0,3672	1,5383		
GAS/ISRE	IFN-stimulated response element	0,6647	1,3257	1,4678		
GATA	GATA transcription factor	0,7793	0,1125	1,2752		
GR/PR	Glucocorticoid receptor/ Progesterone receptor	0,3434	0,2976	1,6046		
HIF	Hypoxia inducible factor	0,1422	0,0536	1,0419		
HNF4	Hepatocyte nuclear factor 4	0,5881	0,0155	0,1271		

L. von Elsner, S. Hagemann, I. Just and A. Rohrbeck
Institute of Toxicology, Hannover Medical School, Hannover, Germany
email: vonelsner.leonie@mh-hannover.de

IRF	Interferon regulatory factor	5,9814	0,1644	2,5214		
MEF2	Myocyte enhancer factor 2	1,8264	0,2365	1,3471		
Myb	v-myb myeloblastosis viral oncogene homolog	10,4140	0,3247	1,2765		
Myc-Max	v-myc myelocytomatosis viral oncogene homolog (avian)	0,9462	0,1391	1,3841		
NF-1	Nuclear factor 1	2,0205	0,1198	0,6820		
NFAT	Nuclear factor of activated T-cells	0,4319	0,0152	8,3213		
NF-E2	Nuclear factor (erythroid-derived 2)	0,3801	0,1923	1,5158		
NFkB	nuclear factor of kappa light polypeptide	5,3229	0,1548	0,2286		
OCT4	POU class 5 homeobox 1	0,1980	0,0438	1,0856		
p53	Tumor protein p53	1,0732	0,1268	0,8805		
Pax-5	Paired box 5	0,0852	0,1248	2,2502		
Pbxl	Pre-B cell leukemia transcription factor-1	0,7122	1,0325	5,3994		
Pit	Pituitary specific transcription factor 1	4,7089	0,0459	3,4729		
PPAR	Peroxisome proliferator-activated receptor	0,9500	0,0175	1,9641		
PXR	Pregnane X receptor	0,1605	0,0056	0,7901		
SMAD	SMAD family	0,6422	0,1353	0,4480		
**Sp1**	**SP1 transcription factor**	**4,5126**	**6,8097**	**2,2478**	**4,5234**	*****
SRF	Serum response factor	0,8572	0,1022	0,2694		
SATB1	Special AT-rich sequence binding protein 1	1	1	1		
Stat1	Signal transducer and activator of transcription 1	0,5694	0,1499	1,9145		
Stat3	Signal transducer and activator of transcription 3	1,6388	0,1156	1,1912		
Stat4	Signal transducer and activator of transcription 4	0,7991	0,0875	0,5500		
Stat5	Signal transducer and activator of transcription 5	0,3658	0,0333	6,9143		
**Stat6**	**Signal transducer and activator of transcription 6**	**0,4820**	**0,0714**	**0,1731**	**0,2422**	*****
TCF/LEF	Runt-related transcription factor 2	0,9588	0,2271	0,6293		
YY1	YY1 transcription factor	13,5452	0,0734	0,1187		
TR	Thyroid hormone receptor	0,2155	0,4007	0,9743		
TFIID	TATA boxbinding protein	0,3157	1,7109	0,7095		

Naunyn-Schmiedeberg's Archives of Pharmacology
C3 exoenzyme impairs cell proliferation and apoptosis by altering the activity of transcription factors
L. von Elsner, S. Hagemann, I. Just and A. Rohrbeck
Institute of Toxicology, Hannover Medical School, Hannover, Germany
email: vonelsner.leonie@mh-hannover.de

Supplementary Material

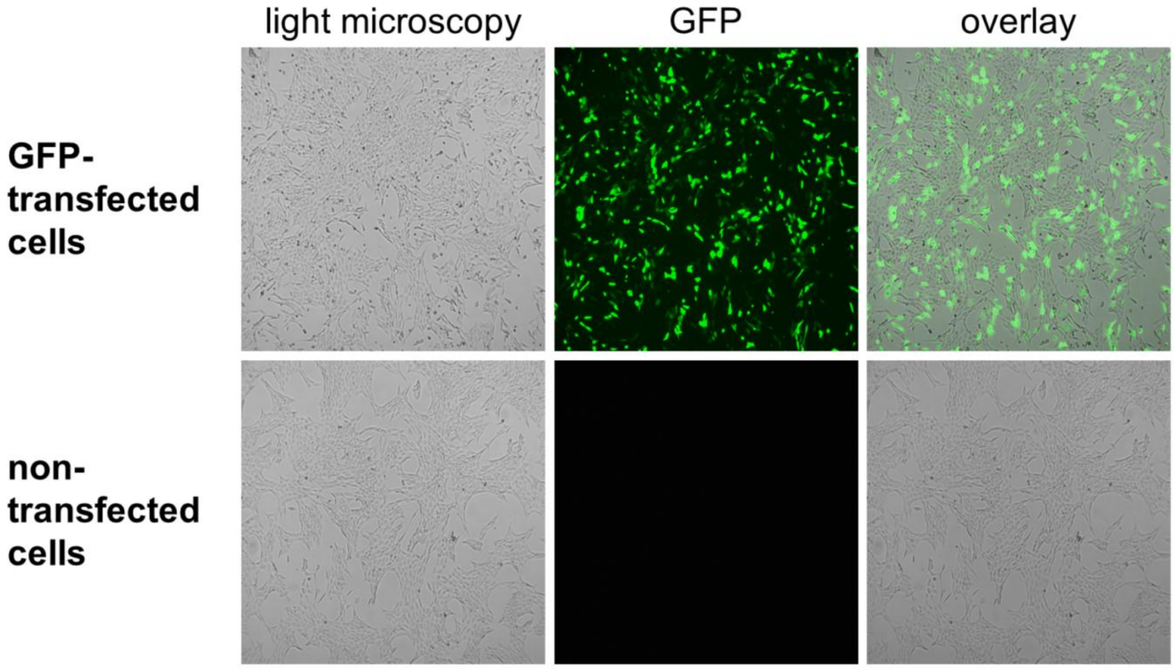

Fig. S1 Effectivity of transfection system. The transfection efficiency was determined by light and fluorescence microscopy (with 4x magnification) of non-transfected cells or cells transfected with a GFP-including construct as positive control

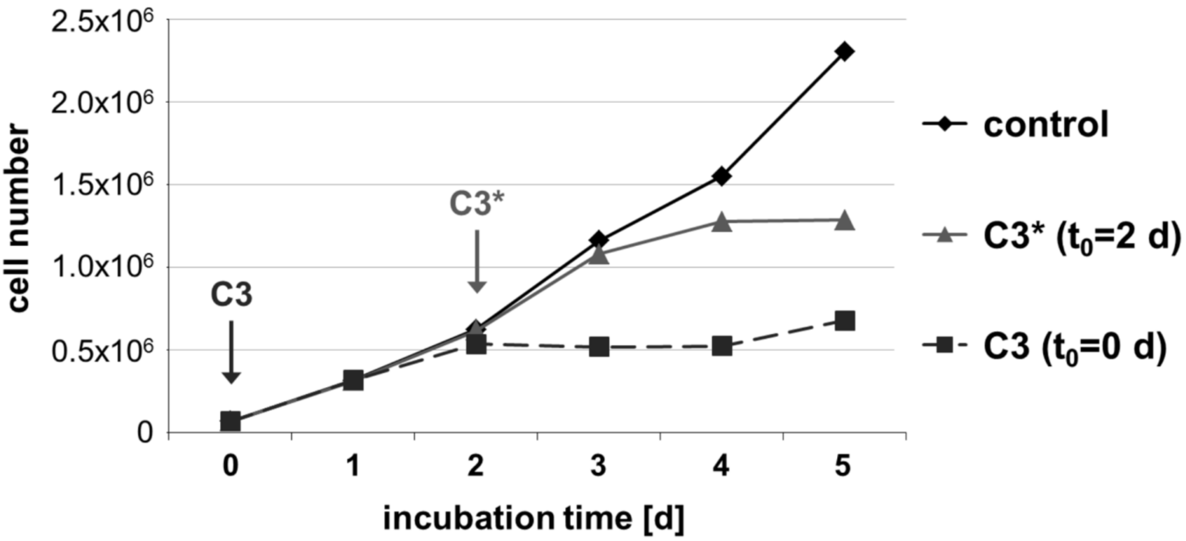

Fig. S2 Time-dependent C3-mediated inhibition of cell proliferation. By use of growth kinetic experiments, the temporal delay of the C3-induced anti-proliferative effect was examined. HT22 cells were started to treat with 500 nM C3 at day 0 (C3 t_0=0 d) or respectively on day 2 (C3* t_0=2 d). Every 48 h the medium including C3 was replaced. At indicated time points the cell number was determined by trypan blue counting assay in duplicate. Growth curves represent mean values

C3 exoenzyme impairs cell proliferation and apoptosis by altering the activity of transcription factors
L. von Elsner, S. Hagemann, I. Just and A. Rohrbeck
Institute of Toxicology, Hannover Medical School, Hannover, Germany
email: vonelsner.leonie@mh-hannover.de

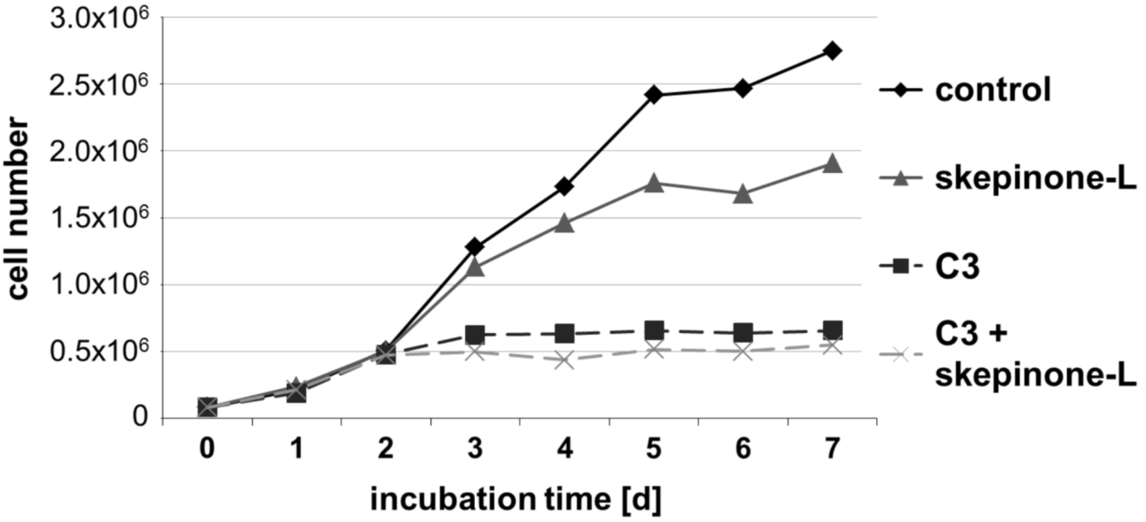

Fig. S3 Skepinone-L inhibits moderately cell proliferation. The impact of 20 nM skepinone-L on cell proliferation was studied by use of growth kinetic experiments. HT22 cells were treated with 500 nM C3, 20 nM skepinone-L or a combination of both. The medium including C3 or skepinone-L was replaced every 48 h. At indicated time points the cell number was determined by trypan blue counting assay in duplicate. Growth curves represent mean values
